# Anatomical Variations That Can Lead to Spine Surgery at the Wrong Level: Part I, Cervical Spine

**DOI:** 10.7759/cureus.8667

**Published:** 2020-06-17

**Authors:** Manan Shah, Dia R Halalmeh, Aubin Sandio, R. Shane Tubbs, Marc D Moisi

**Affiliations:** 1 Neurosurgery, Wayne State University, Detroit Medical Center, Detroit, USA; 2 Neurosurgery, Detroit Medical Center, Detroit, USA; 3 Neurosurgery and Structural & Cellular Biology, Tulane University School of Medicine, New Orleans, USA; 4 Anatomical Sciences, St. George's University, St. George's, GRD; 5 Neurosurgery and Ochsner Neuroscience Institute, Ochsner Health System, New Orleans, USA

**Keywords:** anatomical variations, cervical, cervical spine, congenital, sentinel events, spine, surgery at wrong level

## Abstract

Spine surgery at the wrong level is an adversity that many spine surgeons will encounter in their career, and it falls under the wrong-site surgery sentinel events reporting system. The cervical spine is the second most common location in the spine at which surgery is performed at the wrong level. Anatomical variations of the cervical spine are one of the most important incriminating risk factors. These anomalies include craniocervical junction abnormalities, cervical ribs, hemivertebrae, and block/fused vertebrae. In addition, patient characteristics, such as tumors, infection, previous cervical spine surgery, obesity, and osteoporosis, play an important role in the development of cervical surgery at the wrong level. These were described, and several effective techniques to prevent this error were provided. A thorough review of the English-language literature was performed in the database PubMed between 1981 and 2019 to review and summarize these risk factors. Compulsive attention to these factors is essential to ensure patient safety. Therefore, the surgeon must carefully review the patient’s anatomy and characteristics through imaging and collaborate with radiologists to reduce the likelihood of performing cervical spine surgery at the wrong level.

## Introduction and background

Cervical spine surgery at the wrong level is a burdensome situation both for the patient and the surgeon, as it leads to repeated operation with further risks, damages the doctor-patient relationship, and results in legal actions [1]. It has been characterized as wrong-site surgery, and the Joint Commission on Accreditation of Healthcare Organization (JCAHO) reported that wrong-site surgery was the most common sentinel event in 2008 [1,2]. The cervical spine is the second most common location in the spine at which surgery is performed at the wrong level [3,4], and its incidence ranges from 0.09 to 4.5 per 10,000 surgeries performed. In one survey involved 415 spine surgeons, 217 responded that they had performed, at least once, wrong-level surgery during their career [4]. From an estimated 1,300,00 spine procedures, 418 operations had been performed at the wrong level, with 21% performed on the cervical region, 71% on the lumbar region, and 8% on the thoracic spine [1]. Numerous potential risk factors have been implicated, including emergency surgery, time constraints to complete the procedure, and poor communication between the surgeon and the patient [1]. Importantly, unusual patient characteristics and anatomy are major risk factors and should be taken into consideration when performing spine surgery.

In this literature review, we present several cervical spine anatomical variations and patient characteristics that can potentially lead to surgery at the wrong level, including craniocervical junction abnormalities, cervical ribs, hemivertebra, block/fused vertebra, tumors, infection, previous cervical surgery, obesity, and osteoporosis. Efforts to improve identification of these variants and hence the target level through different imaging modalities are likely to decrease the risk of this pitfall and improve the patient’s safety and outcomes. We also describe several important techniques and preventive measures that can help decrease the risk of this problem.

## Review

Material and methods

Peer-reviewed articles were searched through the PubMed database using the search terms “wrong level surgery”, “cervical spine anomalies”, “craniocervical junction abnormalities”, “atlantooccipital assimilation”, “basilar invagination”, “C1 anterior and posterior arch anomalies”, “os odontoideum”, “cervical rib” “cervical hemivertebra”, “Klippel-Feil syndrome”, “obesity and spine surgery”, “osteoporosis and spine surgery”, until April 1, 1981. The search was conducted over a period of three months between October 2018 and December 2018. The articles then were filtered to include full-text articles. All studies published in the English language reporting cervical spine anatomical variations that can lead to surgery at the wrong level were included. Relevant publications on patient characteristics and anatomical aspects in the cervical region that may potentially lead to this misadventure, as well as surgical, cadaveric, and radiologic studies were also included. Articles that reported unrelated material or none of the previously mentioned correlations, as well as duplications among the database were excluded from the study. Finally, the articles were reviewed, and those most representative were selected.

Results and discussion

More than 11,000 articles were found using the initial search terms noted in the preceding section. Articles ranged from early anatomical description and reports of the cervical anatomical variations to the most recent records about these variants. After filtering and further review, using the inclusion and exclusion measures mentioned in the Material and Methods section, 24 peer-reviewed articles were used for this literature review of cervical spine surgery at the wrong level and the spine anomalies that can cause it. The results were summarized as follows.

Craniocervical Junction Abnormalities

**Figure 1:**
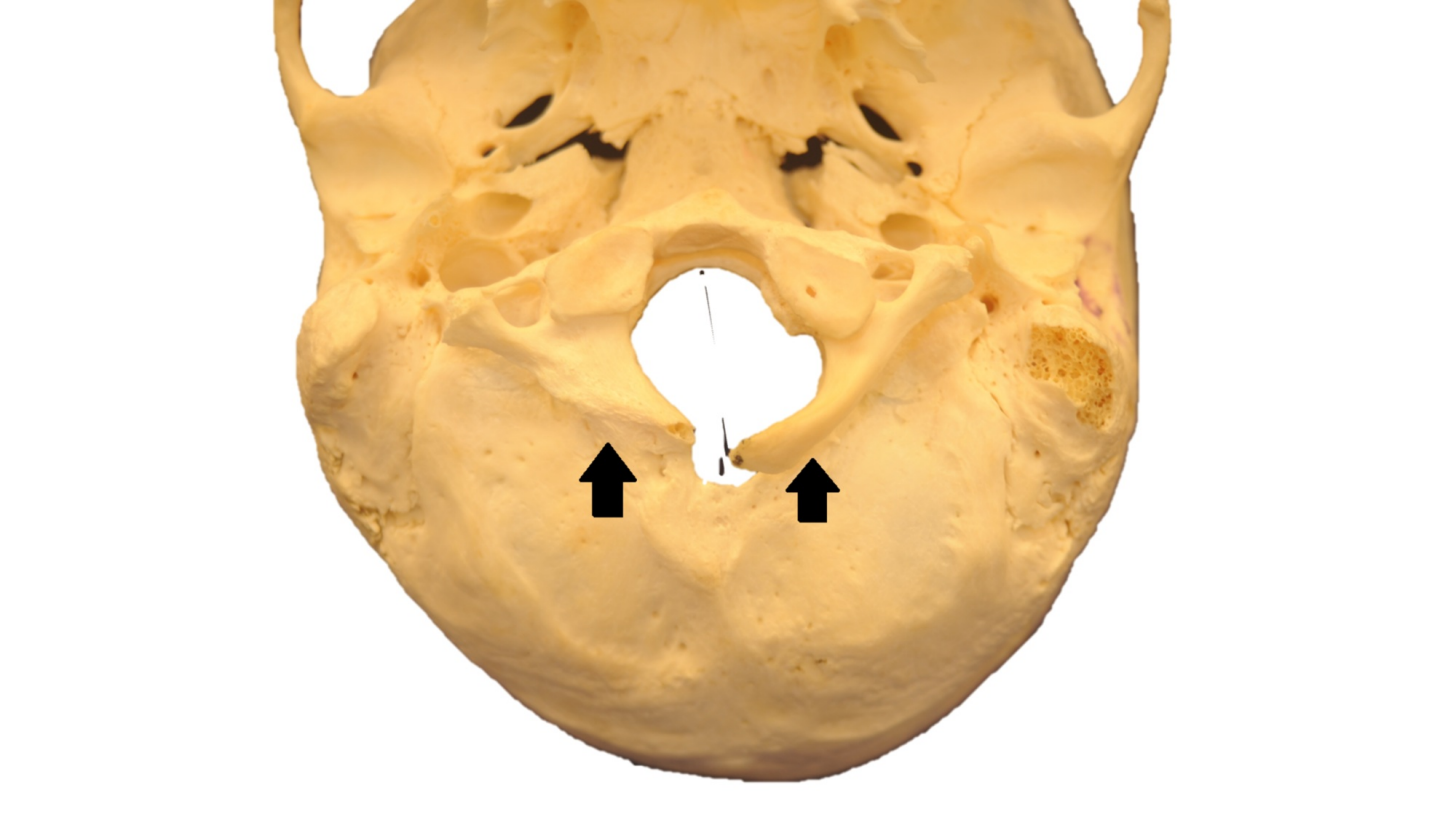
Atlantooccipital assimilation Posteroinferior view of atlantooccipital assimilation (black arrows). It represents the most common congenital abnormality of the upper cervical region with symptoms similar to Klippel-Feil syndrome [7].

Craniocervical junction abnormalities are anatomical variations of the occiput, atlas, and axis, all of which can contribute to surgery at the wrong level. Atlantooccipital assimilation (Figure 1) is the failure of segmentation between the skull and C1, which can be complete or partial [5,6].

**Figure 2:**
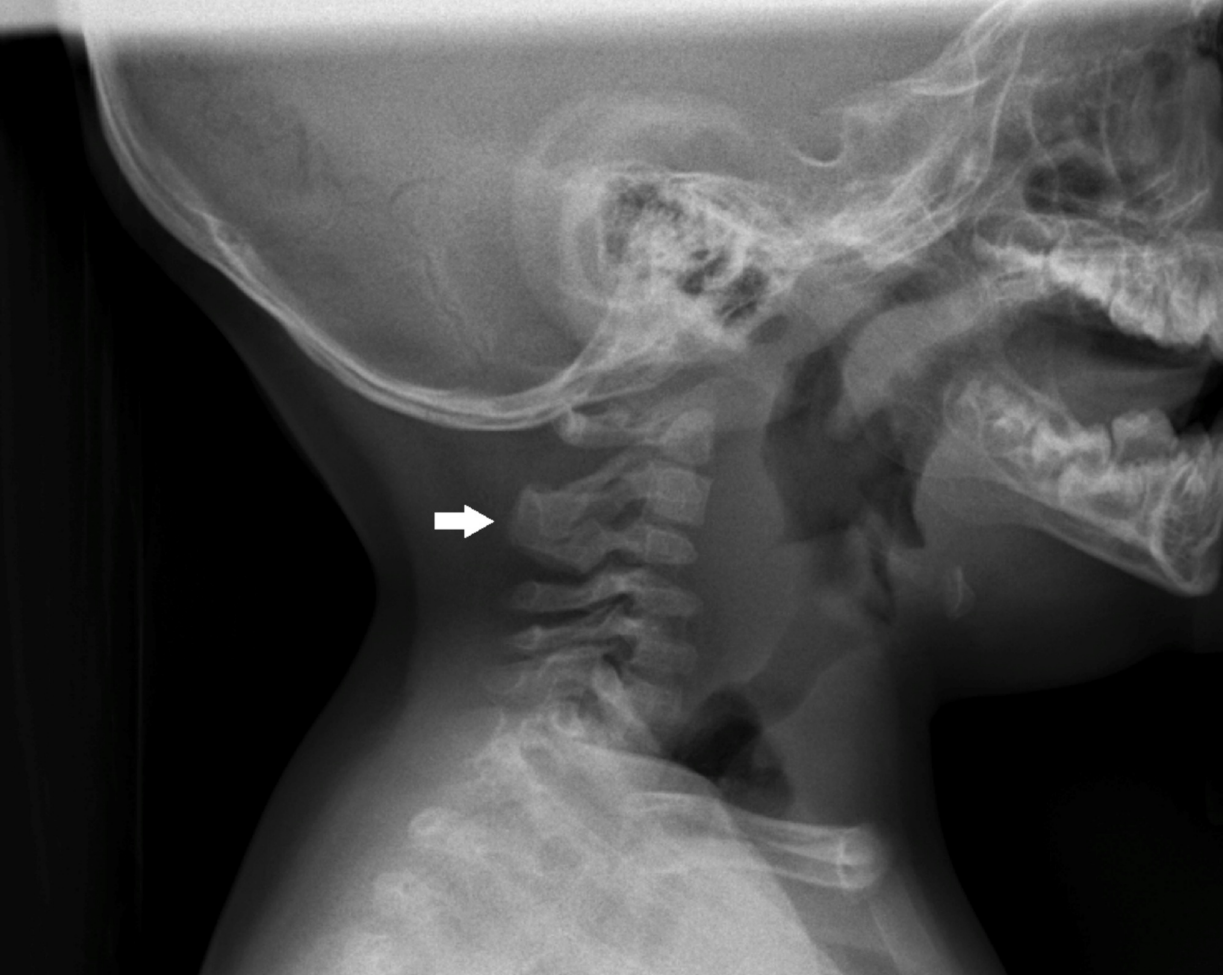
Klippel-Feil syndrome Lateral radiograph of the cervical region showing C2/C3 (white arrow) Klippel-Feil syndrome, a rare congenital disorder characterized by fusion of at least two cervical vertebrae resulting in restricted mobility of the upper neck. Of note, Klippel-Feil syndrome may have signs of basilar invagination [7].

There also is an increased prevalence of associated fusion of the axis and C3 [6]. Basilar invagination is another anomaly that results in the odontoid prolapsing into the foramen magnum [5]. It can be seen in 20% of rheumatoid arthritis patients, but it also presents commonly in other congenital conditions such as Klippel-Feil syndrome (Figure 2), Chiari malformation, Paget’s disease, osteogenesis imperfecta, and syringomyelia [5]. Basilar invagination that is due to secondary causes or congenital conditions is referred to as basilar impression, which is described as softening of the bone at the foramen magnum, and platybasia, in which the base of the skull is flattened [5].

Congenital forms of basilar invaginations and basilar impression are associated with a higher incidence of hindbrain herniation, hypoplasia of the atlas, basiocciput, occipital condyle, platybasia, and atlantooccipital assimilation. It is worth mentioning the craniocervical growth collision theory of Roth as a possible pathological mechanism for basilar invagination. According to Roth, the development of early embryonic neural growth precedes the development of the vertebral column, which grows in a craniocaudal fashion. Reversal of this natural process would result in abnormal formations of the skull base and deformation of the margin of the foramen magnum, occiput, and cervical vertebrae [7]. Basilar invaginations can be categorized into two separate groups, with or without Chiari malformations. Basilar invaginations associated with Chiari malformations can present with chronic cerebellar and vestibular deficits [7]. Additionally, patients are more likely to have a shallow posterior fossa, lower than expected supraocciput and exoccipital heights, lower basioccipital length, and a shorter clivus [7].

**Figure 3:**
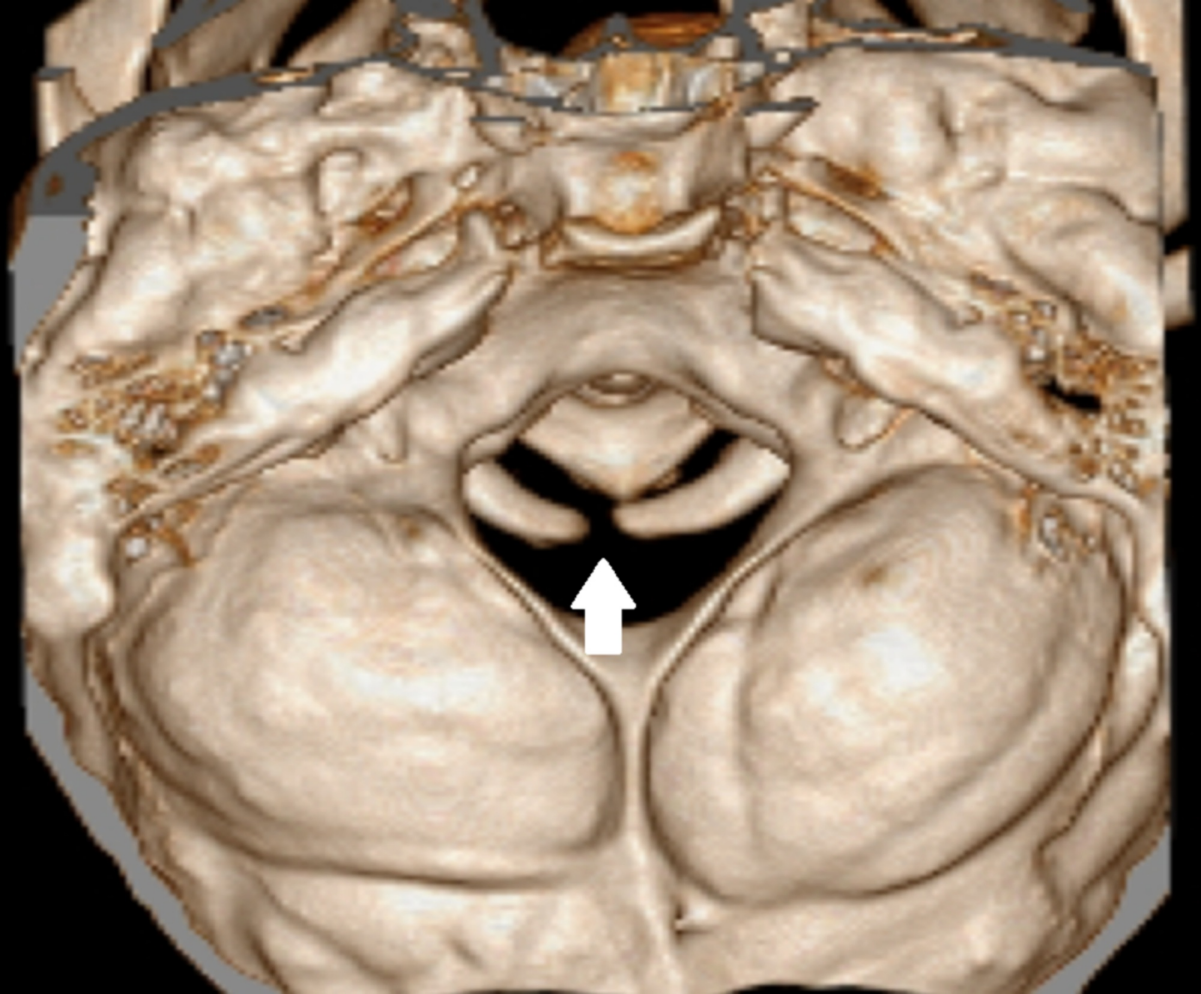
Atlas’ posterior arch defect Three-dimensional CT showing a posterior arch defect of the atlas (white arrow).

The atlas’ posterior arch also can exhibit defects that are believed to be attributable to the failure of local chondrogenesis. These include clefts, hypoplasias, and aplasias. Posterior rachischisis, the most common type of cleft, is seen in 4% of adult autopsy specimens [6]. The vast majority of posterior atlas clefts are midline (97%) [6]. Posterior arch anomalies are divided into five types: A, B, C, D, and E [8]. Type A includes median clefts of the posterior arch, type B includes varying degrees of unilateral defects (Figure 3), type C includes bilateral defects, type D is the absence of the posterior arch with a persistent posterior tubercle, and type E is total agenesis of the posterior arch including the tubercle [8]. Type A occurs in 5.4% of the population, and types B through E occur in 0.69% of the population [8,9]. Anterior arch rachischisis is another type of atlas anomaly that is quite rare and is found in only 0.1% of autopsy specimens [6]. It is often associated with posterior rachischisis, in which case it is referred to as a split atlas [6]. Variants of the odontoid process have also been described, including Bergman ossicle, or persistent ossiculum terminale, a congenital variant of the dens that results from the failure of the terminal ossicle to fuse with the remainder of the odontoid process [6]. This also can be confused with a type 1 odontoid fracture. Os odontoideum is the most common variation of the odontoid process, which refers to an independent osseous structure lying cephalad to the axis body in the location of the odontoid process [6]. Increased awareness of these craniocervical anomalies can prevent cervical spine surgery at the wrong level and may enable early intervention alternatives.

*Cervical Ribs* 

**Figure 4:**
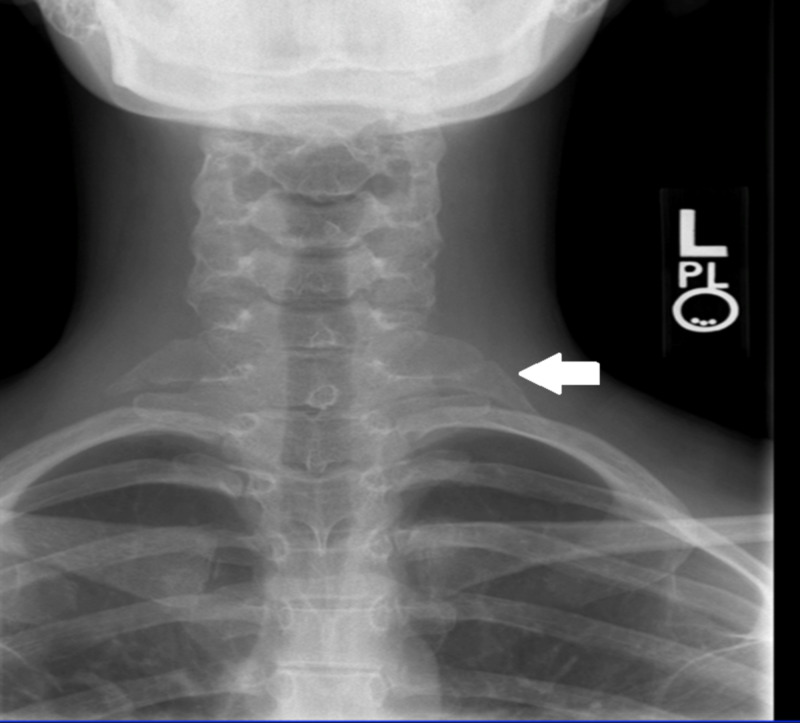
Cervical rib Radiograph of the cervical spine showing a left cervical rib originating at the articulation of C7 and T1 (white arrow).

Cervical ribs are an anatomical anomaly that can lead to inaccurate numerical identification of the level during cervical spine surgery. This anomaly is a supernumerary or extra rib that arises usually from the seventh cervical vertebra [10]. However, these cervical ribs may arise from the fourth, fifth, or sixth cervical vertebra as well [10,11]. There are two types of cervical ribs: complete and incomplete. Complete ribs are those that articulate with the first rib, whereas incomplete ribs are those that end freely in the neck’s soft tissues. Studies have shown that their occurrence varies from 0.58% to 6.2% depending on the population [10]. This anomaly occurs more commonly bilaterally and in females [12], and is believed to be attributable to mutations in the Hox genes [10]. These supernumerary cervical ribs can cause difficulty in identifying the proper level during cervical spine surgery and thus can lead to surgery at the wrong level. X-ray (Figure 4), MRI, or CT can be used in different situations to identify cervical ribs, but the gold standard is a three-dimensional CT.

Hemivertebrae

A hemivertebra is another variant that occurs more commonly in the thoracic spine, but it may also occur in the cervical spine. It is an anomaly in which only half of the vertebral body develops. The incidence is estimated to be approximately 0.3 per 1,000 births and is more common in girls [13,14]. It develops when vertebral body ossification nuclei fail to form and fuse [14]. There are four distinct types of hemivertebrae: incarcerated, nonincarcerated, segmented, and unsegemented [15]. Incarcerated hemivertebrae are those in which the vertebral bodies above and below the abnormal segment accommodate the hemivertebrae, whereas nonincarcerated refers to the failure of accommodation, which results typically in spinal curvature. Segmented, or free, hemivertebrae have a normal disk above and below the defective body and are more likely to lead to progressive curvature, whereas unsegmented hemivertebrae are fused with the vertebral body above and below [15]. Hemivertebra can cause the spine to angle and results in cervical scoliosis. This causes issues in localization during surgery, and care must be taken when performing X-rays. Indeed, some evidence indicates the benefit of obtaining a preoperative CT scan once a hemivertebra is identified, which then can be used as a landmark during cervical spine surgery.

Block Vertebra

**Figure 5:**
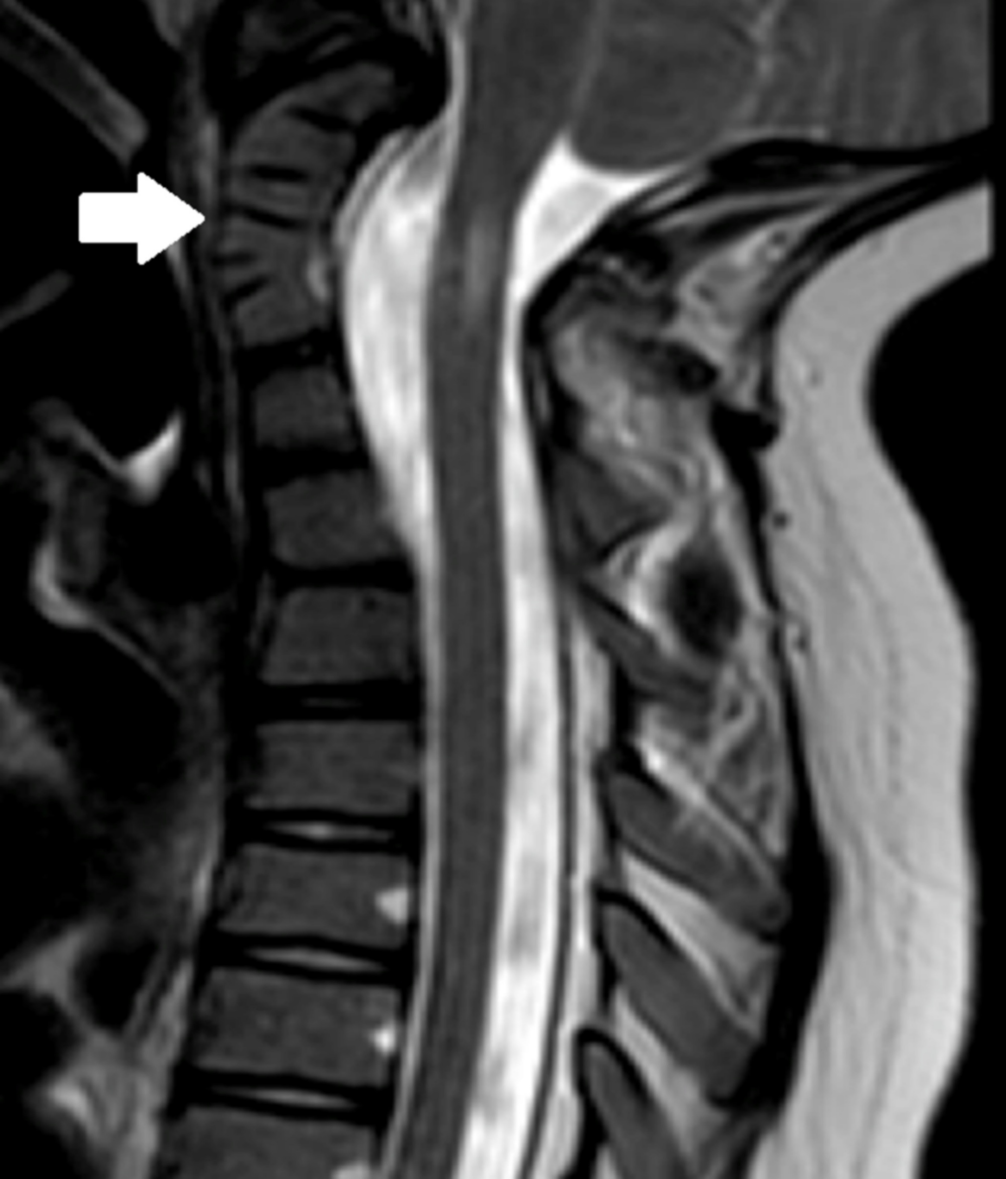
Block vertebrae Sagittal T2-weighted MRI of the cervical spine showing block vertebrae of C3/C4 (white arrow); it may or may not cause hyper- or hypomobility in translation or rotational motion adjacent to the block but can pose a significant challenge to surgery of the cervical spine [18].

Block vertebra is a congenital anomaly that is most common in the cervical and lumbar spine and is attributable to the vertebral column’s improper segmentation [16]. Fusion of adjacent vertebrae occurs through the intervertebral disc and can lead to an abnormal angle in the spine (Figure 5) [16]. In the cervical spine, this segmentation anomaly is referred to as Klippel-Feil syndrome, which is a fusion of two or more vertebrae that often leads to a short neck, low hairline, and limited range of neck motion [17]. This anomaly can cause improper numerical labeling of vertebrae and potential surgery at the wrong level. However, once this variation is identified, it can be used as a landmark to confirm other levels during surgery.

*Tumors and Infection * 

Spinal tumors, particularly metastatic lesions to the spine, can alter the normal spinal anatomy and increase the risk of surgery at the wrong level, and lytic lesions sometimes make it difficult to identify the proper vertebra on X-ray imaging. Infectious lesions of the spine, such as osteomyelitis/diskitis, can also distort the cervical spine’s normal bony anatomy. Chronic infections can destroy the intervertebral disc and cause the vertebral bodies to fuse. In this situation, a detailed preoperative review of imaging is essential to prevent surgery at the wrong level.

Previous Spine Surgery

Previous cervical spine surgery deforms its anatomy, and localizing the correct level for a reoperation is much more difficult as bony defects complicate intraoperative identification of the proper level. In addition, scar tissue makes visual identification more challenging. In patients with prior anterior or posterior cervical instrumentation, X-ray imaging must be reviewed more carefully to identify the anatomy. Ideally, the surgeon should be familiar with the previous surgical procedure to avoid performing surgery at the wrong level.

Obesity and Osteoporosis

Obesity and osteoporosis are comorbid conditions that also may increase the risk of cervical spine surgery at the wrong level. Around 34.9% of U.S. adults, or 78.6 million people, are estimated to be obese, and spine surgeons are operating more on these patients as their incidence rises [19]. The number of spine operations in elderly patients is also increasing, as is the incidence of osteoporosis in spine-surgery patients [20]. There is inadequate radiological exposure in such patients with larger body habitus and decreased bone mineral density [1,21]. For cervical spine surgeries, the shoulders often interfere with X-ray localization, particularly in obese patients. All of these patient characteristics must be identified in advance to avoid surgery at the wrong level.

Strategies to Prevent Cervical Spine Surgery at the Wrong Level

All of these cervical spine anatomical variations and patient characteristics can increase the risk of surgery at the wrong level. The surgeon needs to formulate a reliable routine, which begins in the doctor’s office where he/she must ensure that the patient is seen and consented with the correct side and level(s) [21]. All preoperative imaging, X-rays, CTs, and/or MRIs should be analyzed to identify any anatomical variations. Radiographs of the entire cervical and thoracic spine allow radiologists to confirm the proper level in the cases of supernumerary ribs. Patients who are obese and those with osteoporosis should be kept in mind when preparing for the surgery. In those patients where difficulty in counting levels is anticipated, interventional radiology can place fiducial markers preoperatively [21]. Advanced fiducial markers, including percutaneous fiducial screws and skin adhesive radio-opaque grid lines, have also been shown to improve the accuracy of spinal level localization [3,22,23].

In the operating room, preoperative images should be available at all times to be referred to as necessary. Intraoperative X-rays must be of good quality so that the level(s) of interest can be counted clearly [21]. In order to achieve that, radiologist assistance can be sought to obtain good quality X-rays and identify the correct levels. Moreover, a spinal needle can be inserted systematically between the spinous processes, and a lateral X-ray can help the surgeon navigate to the correct interspace [24]. The shoulders can be taped down, and an assistant can pull the arms during the acquisition of intraoperative X-rays for better visualization of the cervical spine. Other methods to identify the correct level include intraoperative CT, spinal neuronavigation, and transligamentous ultrasound [1,4]. When instrumentation is used, a postoperative X-ray also is recommended to verify the proper placement of instrumentation and the levels, preferably before the incision is closed [21]. All of these strategies are of utmost importance to decrease the risk of cervical spine surgery at the wrong level and allow desired outcomes for the patient.

## Conclusions

Cervical spine surgery at the wrong level is an unfortunate event for surgeons and their patients, as it can lead to further unnecessary surgeries and additional risks for the patient. Although several factors can increase the risk of this surgical error, anatomical variations are one of the major causes. Knowledge of these variants increases the ability of the surgeon to reduce the risk of surgery at the wrong level and provide optimal care for the patient. Finally, cooperating with radiologists can clarify challenging anatomy, and the techniques and the preventive measures mentioned here should be used to prevent cervical spine surgery at the wrong level.
